# The association between TNFR gene polymorphisms and the risk of Hepatitis B Virus-Related Liver Diseases in Chinese population

**DOI:** 10.1038/s41598-018-27623-7

**Published:** 2018-06-18

**Authors:** Liping Ma, Siyuan Chen, Xiaohuan Mao, Yu Lu, Xiaolian Zhang, Xianjun Lao, Xue Qin, Shan Li

**Affiliations:** 1 Department of Clinical Laboratory, Minzu Hospital of Guangxi Zhuang Autonomous Region, Affiliated Minzu Hospital of Guangxi Medical University, Nanning, Guangxi China; 2 Department of Clinical Laboratory, First Affiliated Hospital of Guangxi Medical University, Nanning, Guangxi China

## Abstract

Tumor necrosis factor receptor superfamily 2 (TNFR2) plays an important role in controlling the progression of antiviral and antitumorr. Evidence suggests that TNFR2 is involved in the pathogenesis of HBV-induced liver injury. We therefore examined whether TNFR2 polymorphisms are associated with the risk of HBV-related liver disease in Chinese population. In this case-control study, 115 chronic hepatitis B (CHB) patients, 86 HBV-related liver cirrhosis patients (LC), 272 HBV-related hepatocellular carcinoma patients (HCC) and 269 healthy controls were recruited. TNFR2 rs1061622 and rs1061624 polymorphisms were examined using a polymerase chain reaction-restriction fragment length polymorphism analysis. Binary logistic regression analyses revealed that the A allele of rs1061624 was positively associated with the risk of CHB (AA vs. GG, P = 0.026; AA vs. GA+GG, P = 0.021), LC (AA vs. GG, P = 0.027; AA+GA vs. GG, P = 0.036), and HCC (GA vs. GG, P = 0.046; GA+AA vs. GG, P = 0.031). Moreover, subgroup analysis indicated that male subjects have increased risk in developing CHB and LC. Nevertheless, no association was found between rs1061622 polymorphism and HBV-related liver diseases in the overall or subgroup analyses. Our retrospective study suggests that the TNFR2 rs1061624 polymorphism is associated with HBV-related CHB, LC, and HCC in Chinese population, particularly in males.

## Introduction

Hepatocellular carcinoma (HCC), one of the most widespread and lethal cancers, is ranked as the fifth most common cancer and the third leading cause of cancer-related death. Approximately one million new HCC cases and almost an equal number of deaths are reported annually^1^. HCC is a frequent malignancy in East and Southeast Asia and Sub-Saharan Africa, and it is particularly common in China, which accounts for nearly half of all cases^2,3^.

HCC is a complex and multifactorial process. Environmental factors such as exposure to aflatoxin, excess intake of alcohol, liver cirrhosis, and infections with the hepatitis B virus (HBV) or hepatitis C virus (HCV) have been associated with its development. Chronic infections with either the hepatitis B virus (HBV) or the hepatitis C virus (HCV) account for 75% to 80% of HCC cases^4^. Deficiencies in the immune response to viral infection are likely an important oncogenic factor in chronic viral carriers. If the T-cell response is not strong enough to completely eliminate the viruses from the liver, chronic liver inflammation may be induced. Inflammation has been suggested to be involved in tumorigenesis via promoting angiogenesis, tumor growth, and DNA damage^5–7^.

Tumor necrosis factor alpha (TNF-α), a multifunctional cytokine, has a great influence on host immune response to HBV/HCV infection and the pathogenesis of autoimmune and malignant diseases^8,9^. Specifically, it has been implicated in the pathophysiology of liver cancer^10^. TNF-α mediates its diverse biologic effects through binding to its two cognate cell surface receptors: tumor necrosis factor receptor superfamily 1A (TNFR1A/TNFR1) and tumor necrosis factor receptor superfamily 1B (TNFR1B/TNFR2), both of which are involved in the regulation of several inflammatory pathways through the activation of transcription factor NF-κB. TNFR1, which has a Fas-like “death domain”, is capable of mediating TNF-induced cytotoxicity and TNF-induced cell death. In contrast, the activation of TNFR2 has been shown to be proliferative in hematopoietic cells, particularly T-cells, and it is also known to evoke apoptosis in CD8 cells. In addition, TNFR2 plays a regulatory role in TNF-α’s binding to TNFR1^11^. In general, most of the nucleated cells are capable of producing TNFR1, whereas TNFR2 expression is restricted primarily to cells of hematopoietic lineage. Increased serum levels of the TNF-TNFR superfamily have been reported in patients with solid tumors and liver disease. The expression of the TNF-TNFR superfamily may influence the development and prognosis of various human cancers^12–14^. For example, significantly different expression profiles on the part of TNFR2 have been found in 5-FU-non-responding and -responding liver cancer patients^15^. Moreover, serum concentrations of TNFRs are significantly associated with increased severity in patients with alcoholic liver disease and with mortality in acute alcoholic hepatitis patients^15–17^.

The TNFR2 gene, which was mapped on chromosome 1p36.2, has a polymorphism in exon 6 at codon196 (rs1061622, ATG → AGG). There, a T → G substitution results in a non-conservative amino acid change [methionine (M) → arginine(R)]. This SNP situated at one of four cysteine-rich domains of TNFR2, a site important for optimal TNF binding, has been reported to affect signal transduction and mRNA stability. Signal transduction by the polymorphic allele (196R) may be more efficient than that by the wild-type allele (196 M). Rs1061624, located in the 3′-untranslated region (UTR) of TNFR2 gene, may have functional consequences through its role in signal transduction or mRNA stability^18^.

In addition, TNFR2 gene polymorphism has been associated with a susceptibility to and the progression of various cancers. Such observations have increased our interest in examining the impact of the TNFR2 196 M/R genotype on liver carcinogenesis. To the best of our knowledge, no studies have investigated the association between genetic polymorphisms in TNFR2 and HCC. In the present study, TNFR2 polymorphisms at rs1061622G/T and rs1061624A/G were considered as candidates for investigation in 742 Chinese people. Meanwhile, differences in genotype frequency between the chronic hepatitis (CHB), liver cirrhosis (LC), and hepatocellular carcinoma (HCC) cases and healthy controls were examined.

## Results

### Characteristics of the study populations

The demographic features of the cases and controls enrolled in this study are represented in Table 1. As compared with the control group, the three case groups (CHB, LC, and HCC) have statistically different laboratory results for sex, age, and ethnicity (P < 0.05), the CHB group had a higher proportion of alcohol drinkers, and the LC group had a higher proportion of smokers. There were no significant differences in BMI.

**Table 1:** Demographic and clinical characteristics of the study subjects.

Variables	Controls	CHB		LC		HCC	
	n = 269	n = 115	P value	n = 86	P value	n = 272	P value
Age (years) mean ± SD	46.62 ± 7.04	38.55 ± 12.00	0.000	50.36 ± 12.24	0.000	49.24 ± 11.30	0.001
BMI	22.43 ± 3.41	22.25 ± 3.54	0.467	22.64 ± 4.07	0.642	22.88 ± 13.08	0.256
Gender, N (%)							
Male	150 (55.8)	89 (77.4)	0.000	66 (76.7)	0.001	249 (91.2)	0.000
Female	119 (44.2)	26 (22.6)		20 (23.3)		24 (8.8)	
Smoking, N (%)							
Yes	88 (32.7)	49 (42.6)	0.064	41 (47.7)	0.012	89 (32.7)	0.999
No	181 (67.3)	66 (57.4)		45 (52.3)		183 (67.3)	
Alcohol consumption, N (%)							
Yes	78 (29.0)	57 (49.6)	0.000	30 (34.9)	0.302	88 (32.4)	0.427
No	191 (71.0)	58 (50.4)		56 (65.1)		184 (67.6)	
Ethnicity, N (%)							
Zhuang	137 (50.9)	44 (38.3)	0.044	32 (37.2)	0.037	110 (40.4)	0.000
Han	117 (43.5)	66 (57.4)		51 (59.3)		155 (57.0)	
Others	15 (5.6)	5 (4.3)		3 (3.5)		2 (2.6)	

CHB = chronic hepatitis B, LC = liver cirrhosis, HCC = hepatocellular carcinoma, BMI = body mass index, SD = standard deviation.

### Risk assessment between TNFR2 genotypes and CHB

The genotype frequency of TNFR2 gene rs1061622 and rs1061624 polymorphisms is listed in Table 2. In the control group, the genotype distributions of rs1061624 were not consistent with the Hardy-Weinberg assumption. We then performed a logistic regression analysis (adjusting for gender, age, ethnicity, smoking, alcohol consumption, and BMI) and found that there was a significant association between TNFR2 rs1061624 polymorphism and CHB susceptibility in the additive model (AA vs. GG) and the recessive model (AA vs. GA+GG), with adjusted ORs of 2.666 (95% CI = 1.126−6.316, P = 0.026) and 2.459 (95% CI = 1.145−5.281, *P* = 0.021), respectively. However, the *TNFR2* rs1061622 polymorphism did not influence susceptibility to CHB in any of the genetic models (additive genetic models: GG vs. TT and TG vs. TT, recessive genetic model: GG vs. TG+TT, and dominant genetic model: GG+TG vs. TT).

**Table 2:** Genotype Distributions and Allele Frequencies of TNFR2 Polymorphisms Between Cases and Controls.

Model	Controls	CHB	LC	HCC	CHB vs Controls		LC vs Controls		HCC vs Controls	
	n = 269 (%)	n = 115 (%)	n = 86 (%)	n = 272 (%)	R(95%CI)*	P*	R(95%CI)*	P*	R(95%CI)*	P*
rs1061622										
T	460 (85.5)	203 (88.3)	151 (87.8)	469 (86.2)	Reference		Reference		Reference	
G	78 (14.5)	27(11.7)	21 (12.2)	75 (13.8)	0.775 (0.453–1.325)	0.352	0.893 (0.522–1.529)	0.680	0.964 (0.661–1.407)	0.850
TT	196 (72.9)	90 (78.3)	66 (76.7)	200 (73.5)	Reference		Reference		Reference	
TG	68 (25.3)	23 (20.0)	19 (22.1)	69 (25.4)	0.721 (0.388–1.307)	0.273	0.884 (0.480–1.628)	0.692	0.970 (0.631–1.492)	0.890
GG	5 (1.9)	2 (1.7)	1 (1.2)	3 (1.1)	1.001 (0.118–8.519)	0.999	0.851 (0.090–7.368)	0.855	0.870 (0.176–4.289)	0.864
Dominant model										
TT	196 (72.9)	90 (78.3)	66 (76.7)	200 (73.5)	Reference		Reference		Reference	
TG + GG	73 (27.2)	25 (21.7)	20 (23.3)	72 (26.5)	0.725 (0.400–1.314)	0.289	0.880 (0.483–1.601)	0.675	0.965 (0.632–1.471)	0.867
Recessive model										
TT + TG	264 (98.1)	113 (98.3)	85 (98.8)	269 (98.9)	Reference		Reference		Reference	
GG	5 (1.9)	2 (1.7)	1 (1.2)	3 (1.1)	1.104 (0.131–9306)	0.928	0.845 (0.094–7.593)	0.880	0.877 (0.178–4.307)	0.871
rs1061624										
G	324 (60.2)	120 (52.6)	87 (51.2)	304 (55.7)	Reference		Reference		Reference	
A	214 (39.8)	108 (47.4)	83 (48.8)	242 (44.3)	1.363 (0.949–1.959)	0.094	1.432 (0.995–2.060)	0.053	1.272 (0.973–1.662)	0.078
GG	76 (28.3)	27 (23.7)	14 (16.5)	60 (22.0)	Reference		Reference		Reference	
GA	172 (63.9)	66 (57.9)	59 (69.4)	184 (67.4)	1.128 (0.625–2.038)	0.689	1.895 (0.972–3.693)	0.06	1.563 (1.009–2.422)	0.046^#^
AA	21 (7.8)	21 (18.4)	12 (14.1)	29 (10.6)	2.666 (1.126–6.316)	0.026^#^	2.947 (1.130–7.689)	0.027^#^	1.955 (0.939–4.073)	0.073
Dominant model										
GG	76 (28.3)	27 (23.7)	14 (16.5)	60 (22.0)	Reference		Reference		Reference	
GA + AA	193 (71.7)	87 (76.3)	72 (83.5)	212 (78)	1.314 (0.742–2.329)	0.349	2.016 (1.046–3.887)	0.036^#^	1.605 (1.043–2.470)	0.031^#^
Recessive model										
GG + GA	248 (92.8)	94 (81.6)	74 (85.9)	243 (89.4)	Reference		Reference		Reference	
AA	21 (7.8)	21 (18.4)	12 (14.1)	29 (10.6)	2.459 (1.145–5.281)	0.021^#^	1.830 (0.817–4.099)	0.142	1.411 (0.703–2.728)	0.305

*Adjusted for age, gender, ethnicity, smoking, alcohol consumption,and BMI.

^#^The data reach statistical significance.

### Risk assessment between TNFR2 genotypes and LC

After adjusting for the demographic and clinical factors, variants of TNFR2 rs1061624 were found to be associated with LC development. Patients with the AA genotype and at least one copy of the A allele (dominant model) were found to be at more than double the normal risk of LC (ORs = 2.947 and 2.016, respectively; P < 0.05). In contrast, the variants of TNFR2 rs1061622 were not found to be associated with LC risk.

### Risk estimation between TNFR2 genotypes and HCC

In our binary logistic regression analyses, the data showed that individuals with the TNFR2 rs1061624 GA genotype had an approximately 50% increased risk of HCC (OR = 1.563, 95% CI = 1.009−2.422, *P* = 0.046) after adjusting for age, gender, smoking, alcohol consumption, and BMI. Furthermore, the data in the dominant model (GA+AA vs. GG) also showed a significantly increased risk of HCC among such individuals (OR = 1.605, 95% CI = 1.043−2.470, P = 0.031). However, the present data revealed that the TNFR2 rs1061622 polymorphism was not associated with HCC risk.

### Risk estimation between TNFR2 genotypes and HBV-related diseases according to gender

The gender-stratification of TNFR2 polymorphisms indicated that their implications were mostly restricted to male subjects (Table 3). For rs1061624, significantly increased CHB risk was found among male subjects in the allele model A vs. G (OR = 1.668, 95% CI = 1.056–2.634, *P* = 0.022), the additive model AA vs. GG (OR = 4.280, 95% CI = 1.405–13.041, P = 0.026), and the recessive model AA vs. AG+GG (OR = 3.032, 95% CI = 1.146−8.021, P = 0.025). A significantly increased LC risk was found among male subjects in the additive model AA vs. GG (OR = 3.361, 95% CI = 1.114−10.146, *P* = 0.031). In contrast, the present data indicate no differences in the genotype distributions of TNFR2 rs1061622 among male and female subjects (data not shown).

**Table 3:** Risk estimation between TNFR2 genotypes and HBV-related diseases in males.

SNP	CHB vs Controls		LC vs Controls		HCC vs Controls	
	R(95%CI)*	P*	R(95%CI)*	P*	R(95%CI)*	P*
rs1061622						
T	Reference		Reference		Reference	
G	0.718 (0.362–1.421)	0.341	0.778 (0.392–1.545)	0.474	1.043 (0.684–1.591)	0.845
TT	Reference		Reference		Reference	
TG	0.698 (0.327–1.488)	0.351	0.656 (0.301–1.430)	0.289	1.108 (0.687–1.787)	0.647
GG	0.374 (0.007–20.029)	0.629	1.878 (0.154–22.876)	0.621	0.625 (0.085–4.585)	0.644
Dominant model						
TT	Reference		Reference		Reference	
TG + GG	0.688 (0.342–1.461)	0.331	0.695 (0.325–1.483)	0.246	1.082 (0.677–1.731)	0.742
Recessive model						
TT+TG	Reference		Reference		Reference	
GG	0.431 (0.008–23.782)	0.681	2.129 (0.352–1.483)	0.346	0.610 (0.084–4.452)	0.626
rs1061624						
G	Reference		Reference		Reference	
A	1.668 (1.056–2.634)	0.028^#^	1.492 (0.972–2.288)	0.067	1.268 (0.943–1.705)	0.116
GG	Reference		Reference		Reference	
GA	1.649 (0.767–3.547)	0.200	1.747 (0.824–3.703)	0.146	1.548 (0.966–2.479)	0.069
AA	4.280 (1.405–13.041)	0.011^#^	3.361 (1.114–10.146)	0.031^#^	1.882 (0.831–4.267)	0.130
Dominant model						
GG	Reference		Reference		Reference	
GA + AA	1.953 (0.928–4.108)	0.078	0.519 (0.249–1.084)	0.081	1.584 (0.997–2.517)	0.052
Recessive model						
GG+GA	Reference		Reference		Reference	
AA	3.032 (1.146–8.021)	0.025^#^	2.258 (0.867–5.878)	0.095	1.381 (0.653–2.920)	0.398

*Adjusted for age, gender,ethnicity, smoking and alcohol consumption, and BMI.

^#^The data reaches a statistical significant level.

## Discussion

HBV is a prototype member of the Hepadnaviridae family of viruses. These viruses can induce chronic and persistent infections accompanied by immune-mediated liver injury in the infected host. Persistent HBV infection is a major risk factor for the development of hepatic decompensation, cirrhosis, and in particular, hepatocellular carcinoma^19^. Hepatitis B is among the most important infectious diseases in China. More than 120 million people are reportedly to be HBV carriers. An estimated 20 million suffer from CHB, and almost 300,000 die annually from the chronic consequences of HBV infection, such as HBV-related LC or HCC^20^. To our knowledge, this is the first study to investigate the association between SNPs of TNFR2 and the presence of CHB, HBV-related LC, and HBV-related HCC in a Chinese population. We demonstrate, for the first time, that rs1061624 in TNFR2 is strongly associated with an increased risk of CHB, LC, and HCC. Nevertheless, such an effect was not observed for rs1061622.

The pathogenesis of HBV is complex. HBV itself is not directly cytotoxic, and most of the liver injury is mediated by the host immune response^9^. TNF-α plays a major role in the pathogenesis of HBV-induced liver injury. It can compromise immune-mediated virological control and further cause collateral hepatocyte damage and cirrhosis and potentially promote hepatocellular carcinoma. Several studies have described high levels of TNF-α in patients with HBV infection^9^. The pleiotropic effects were created via the TNFR1 and TNFR2 hepatocyte membrane receptors. TNFR2, identified as a survival factor for the immune cell subset, has been reported in the context of inflammation. Lacking or overexpressing TNFR-2 results in a severe inflammatory syndrome^21,22^. TNFR2 signaling has been found to have an effect on anti-viral and anti-tumor activity in hepatocytes and myeloid cells. Ping *et al*.^23^ reported that TNFR2 is associated with heart failure risk in type 2 diabetes mellitus patients. Ham *et al*.^12^ found that TNFR2 plays distinct roles in the response of the hepatic microenvironment to tumor cell entry into the liver, with TNFR2 knockout being detrimental to the growth of liver metastases. According to Heemann *et al*.^13^, elevations of circulating levels of TNFR and high circulating levels of soluble TNFR2 are negative predictors of treatment response in T-cell non-Hodgkin lymphomas.

Given that TNFR2 plays an important role in inflammation and carcinogenesis, SNPs in TNFR2 may affect cell phenotypes and cause the risk of developing inflammation-related diseases. SNPs in TNFR2 have been associated with susceptibility to Human T-cell lymphotropic virus type I, which is associated myelopathy breast cancer^24^ and systemic lupus erythematosus^25^, and SNPs in TNFE2 have been also associated with survival in breast cancer^26^ and non-small cell lung cancer cases^27^. Only one study to date has examined the association between TNFR2 polymorphisms and the risk of liver disease. Nguyen *et al*.^28^ investigated the influence of TNFR2 T676G polymorphisms on severe acute alcoholic hepatitis. The findings of these researchers did not support an association.

HBV-related diseases are more common in males than females. In the present study, we observed a gender difference regarding the degree to which the TNFR2 rs1061624 polymorphism affects CHB and LC risk. Males who carried the rs1061624 AA genotype were at a significantly higher risk of CHB and LC. In contrast, these differences were not observed in females. The mechanisms underlying this gender difference remain unknown. The potentially elevated risk in males may be due to the small number of patients examined in our series. On the other hand, males’ increased exposure to toxic effects (such as tobacco and alcohol abuse) may account for the problem.

In summary, the data presented in this study show, for the first time, that rs1061624 polymorphism in TNFR2 is associated with CHB, LC,and HCC in Chinese patient population with HBV, particularly for males. Detection of this polymorphism could serve as a potential marker in prediction of risk of development of HBV-related diseases. However, these observations were based on a genetic analysis of a single gene using a relatively small sample of patients with HBV-related diseases. Thus, the results should be further validated using an independent prospective clinical study.

## Materials and Methods

### Study population

Among the patients with chronic hepatitis B virus infection who were admitted to the First Affiliated Hospital of Guangxi Medical University from April 2014 to October 2014, 473 patients were selected. One hundred and fifteen had CHB, 86 had HBV-induced liver cirrhosis (LC) and 272 had HCC. All of the patients were positive for HBsAg, anti-HBc, and hepatitis B e antigen (HBeAg) or hepatitis B e antibody (HBeAb) for at least 6 months. The diagnoses of individuals were based on a thorough history, clinical examination, and laboratory evaluation.

CHB was defined as (i) being HBsAg-positive for at least six months and (ii) having serum levels of ALT or AST is greater than 40 IU/mL. LC was defined based on (i) clinical symptoms and characteristic, (ii) pathologic exams or typical morphologic findings upon computed tomography (CT) or ultrasonography, and (iii) laboratory features. HBV-HCC subjects were confirmed using histological or cytological methods or AFP levels higher than 400 ng/mL that were associated with at least one positive imaging result, such as computed tomography (CT), magnetic resonance imaging (MRI) or ultrasonography. During the same study period, 269 unrelated healthy controls who visited the general health check-up centers at the same hospitals were also enrolled. The inclusion criterion for the controls was the absence of any evidence of a prior history of cancer or any other serious illness.

Demographic data from each individual, such as gender, age, body mass index (BMI), ethnicity, smoking status, and alcohol consumption status were collected. All individuals were Chinese and were from Guangxi district. Informed consent was obtained from each individual regarding the use of their DNA. The ethics committee of the First Affiliated Hospital of Guangxi Medical University approved the study protocol. Additionally, all of the methods in this study were carried out in accordance with the principles of the Declaration of Helsinki.

### DNA extraction

Genomic DNA was extracted from peripheral blood mono-nuclear cells (sampled in an EDTA tube) using the QIAamp DNA blood mini kit as described by the manufacturer (Qiagen, Hilden, Germany) and stored at −80 °C until use.

### Genotype analysis

**Figure 1:**
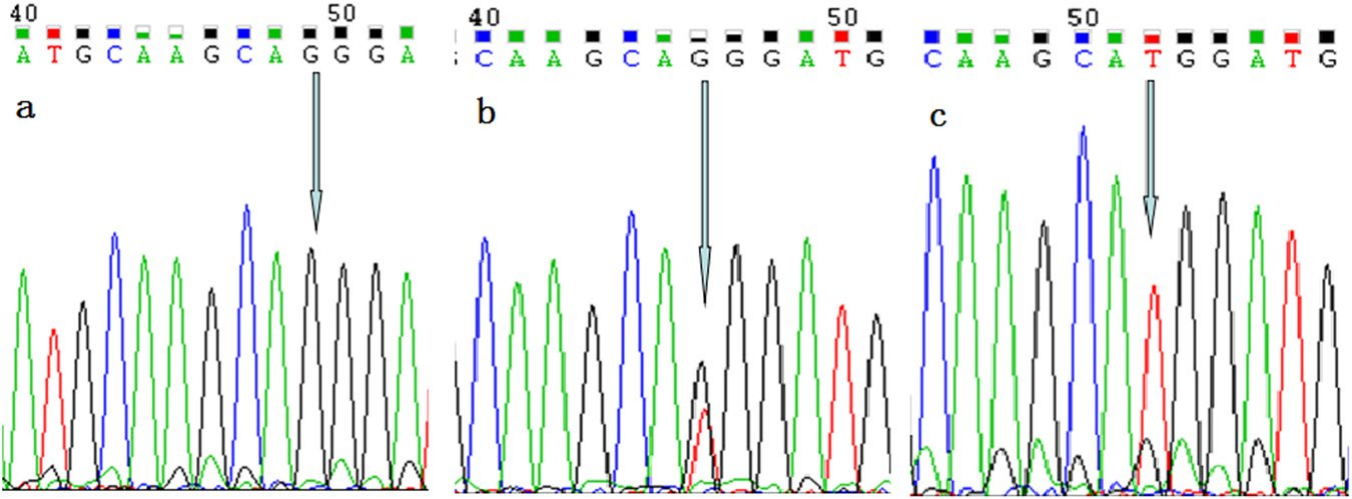
Sequencing map of the genotype for the TNFR2 rs1061622 polymorphism. Arrow in parts (**a**–**c**) indicates GG, G/T and TT genotypes, respectively.

**Figure 2:**
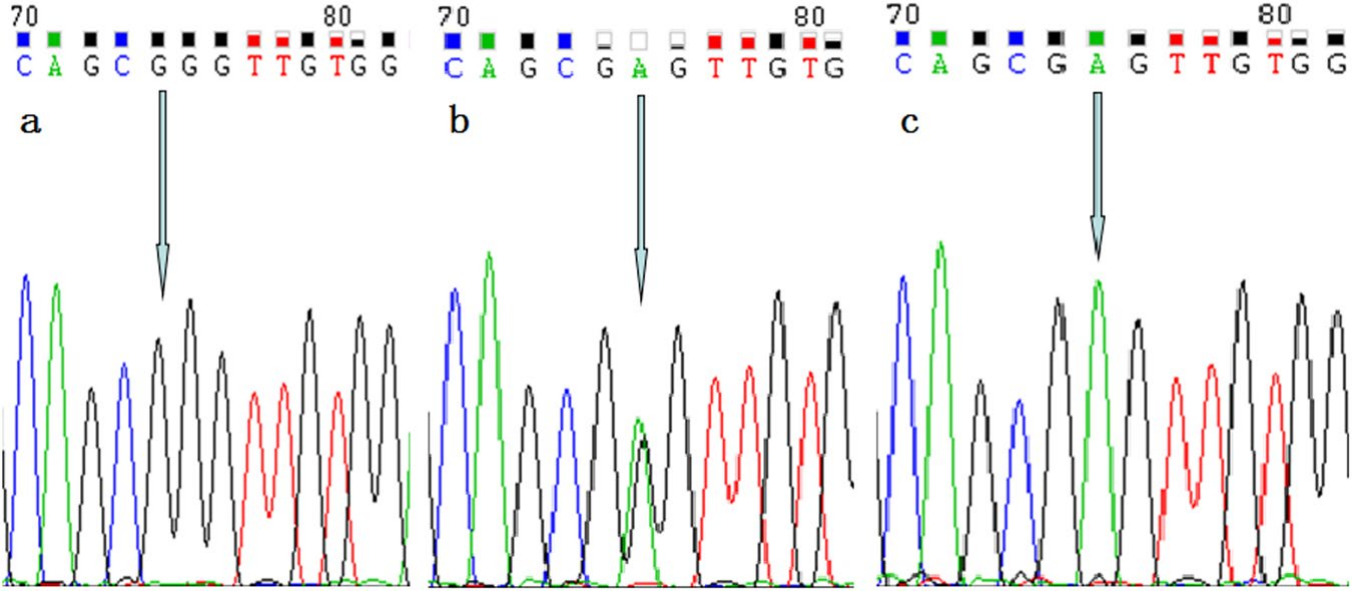
Sequencing map of the genotype for the TNFR2 rs1061624 polymorphism. Arrow in parts (**a**–**c**) indicates GG, G/A, and AA genotypes, respectively.

TNFR2 gene polymorphisms (rs1061622G/T and rs1061624A/G) were examined using polymerase chain reaction-restriction fragment length polymorphism (PCR-RFLP) analysis. The primer sequences, the restriction enzymes used, and the length of the resulting polymerase chain reaction products are given in Table 4. In addition, to validate the results of the genotyping assays, 10% of the PCR-amplified DNA samples were randomly selected and examined via DNA sequencing in an ABI PRISM 3730 (Figs 1 and 2). The results were 100% concordant.

**Table 4:** Primer Sequence and the Reaction Condition for Genotyping TNFR2 Polymorphisms.

SNP	Primer Sequence	Annealing temperature	Product size (bp)	Restriction enzyme
rs1061622	Forward:5′-TCCTCCAGCTGTAACGTGG-3′	63 °C	TT:246 bp, 45 bp	Hin1II
	Reverse:5′-GACAGGCAGACAGAAGGAGT-3′		GG:291 bp	
			TG:291 bp, 246 bp, 45 bp	
rs1061624	Forward:5′-TGGGCCAAGTTCCTCTAGTG-3′	58 °C	GG:71 bp, 106 bp	MspA1I
	Reverse:5′-CAGGTCACAGAGAGTCAGGG-3′		AA:177 bp	
			GA:177 bp, 71 bp, 106 bp

### Statistical analysis

Statistical analyses were conducted using SPSS 16.0 software. The demographic and clinical data were compared between groups via a one-way ANOVA test for continuous variables and a chi-squared test or Fisher’s exact test (when the expected number in any cell was less than five) for categorical variables. The odds ratio (OR) with its corresponding 95% confidence interval (95% CI) were used to assess the strength of each association. Logistic regression analysis was used to adjust the statistical findings for age, gender, BMI, ethnicity, smoking status, and alcohol consumption status. The probability of Hardy-Weinberg equilibrium (HWE) in each group was tested using the chi-squared test. All statistical tests listed above were two tailed, and p < 0.05 was considered statistically significant.

### Data availability

The datasets generated and analyzed during the current study are available from the corresponding authors on reasonable request.

## References

[1] Siegel RL, Miller KD, Jemal A (2017). Cancer Statistics, 2017. CA: a cancer journal for clinicians.

[2] Chen W (2016). Cancer statistics in China, 2015. CA: a cancer journal for clinicians.

[3] Yang JD, Roberts LR (2010). Hepatocellular carcinoma: A global view. Nature reviews. Gastroenterology & hepatology.

[4] Dragani TA (2010). Risk of HCC: genetic heterogeneity and complex genetics. Journal of hepatology.

[5] Molinaro, R. *et al*. Inflammation and Cancer: In Medio Stat Nano. *Current medicinal chemistry* (2017). 10.2174/0929867324666170920160030 PMC5860929 28933296

[6] Balkwill F, Mantovani A (2001). Inflammation and cancer: back to Virchow?. Lancet.

[7] Grivennikov SI, Karin M (2011). Inflammatory cytokines in cancer: tumour necrosis factor and interleukin 6 take the stage. Annals of the rheumatic diseases.

[8] Laidlaw SM (2017). Tumor Necrosis Factor Inhibits Spread of Hepatitis C Virus Among Liver Cells, Independent From Interferons. Gastroenterology.

[9] Valaydon Z (2016). The role of tumour necrosis factor in hepatitis B infection: Jekyll and Hyde. Clinical & translational immunology.

[10] Huang BP, Lin CS, Wang CJ, Kao SH (2017). Upregulation of heat shock protein 70 and the differential protein expression induced by tumor necrosis factor-alpha enhances migration and inhibits apoptosis of hepatocellular carcinoma cell HepG2. International journal of medical sciences.

[11] MacEwan DJ (2002). TNF receptor subtype signalling: differences and cellular consequences. Cellular signalling.

[12] Ham B (2015). TNF Receptor-2 Facilitates an Immunosuppressive Microenvironment in the Liver to Promote the Colonization and Growth of Hepatic Metastases. Cancer research.

[13] Heemann C (2012). Circulating levels of TNF receptor II are prognostic for patients with peripheral T-cell non-Hodgkin lymphoma. Clinical cancer research: an official journal of the American Association for Cancer Research.

[14] Gupta R, Sharma SC, Das SN (2008). Association of TNF-alpha and TNFR1 promoters and 3′ UTR region of TNFR2 gene polymorphisms with genetic susceptibility to tobacco-related oral carcinoma in Asian Indians. Oral oncology.

[15] Matsuyama R (2006). Predicting 5-fluorouracil chemosensitivity of liver metastases from colorectal cancer using primary tumor specimens: three-gene expression model predicts clinical response. International journal of cancer. Journal international du cancer.

[16] Spahr L (2004). Soluble TNF-R1, but not tumor necrosis factor alpha, predicts the 3-month mortality in patients with alcoholic hepatitis. Journal of hepatology.

[17] Naveau S (1998). Plasma levels of soluble tumor necrosis factor receptors p55 and p75 in patients with alcoholic liver disease of increasing severity. Journal of hepatology.

[18] Puga I (2005). A polymorphism in the 3′ untranslated region of the gene for tumor necrosis factor receptor 2 modulates reporter gene expression. Endocrinology.

[19] Stasi C, Silvestri C, Voller F (2017). Emerging Trends in Epidemiology of Hepatitis B Virus Infection. Journal of clinical and translational hepatology.

[20] Liang X (2013). Reprint of: Epidemiological serosurvey of Hepatitis B in China–declining HBV prevalence due to Hepatitis B vaccination. Vaccine.

[21] Peschon JJ (1998). TNF receptor-deficient mice reveal divergent roles for p55 and p75 in several models of inflammation. Journal of immunology (Baltimore, Md. : 1950).

[22] Douni E, Kollias G (1998). A critical role of the p75 tumor necrosis factor receptor (p75TNF-R) in organ inflammation independent of TNF, lymphotoxin alpha, or the p55TNF-R. The Journal of experimental medicine.

[23] Ping Z, Aiqun M, Jiwu L, Liang S (2017). TNF Receptor 1/2 Predict Heart Failure Risk in Type 2 Diabetes Mellitus Patients. International heart journal.

[24] Nishimura M (2000). Tumor necrosis factor, tumor necrosis factor receptors type 1 and 2, lymphotoxin-alpha, and HLA-DRB1 gene polymorphisms in human T-cell lymphotropic virus type I associated myelopathy. Human immunology.

[25] Horiuchi T (2007). A functional M196R polymorphism of tumour necrosis factor receptor type 2 is associated with systemic lupus erythematosus: a case-control study and a meta-analysis. Annals of the rheumatic diseases.

[26] Mestiri S, Bouaouina N, Ben Ahmed S, Chouchane L (2005). A functional polymorphism of the tumor necrosis factor receptor-II gene associated with the survival and relapse prediction of breast carcinoma. Cytokine.

[27] Guan X (2011). TNFRSF1B +676 T>G polymorphism predicts survival of non-small cell lung cancer patients treated with chemoradiotherapy. BMC cancer.

[28] Nguyen-Khac E (2010). Lack of association between tumour necrosis factor receptor types 1 and 2 gene polymorphism and severe acute alcoholic hepatitis. European journal of gastroenterology & hepatology.

